# Peer Review of “Development of a Conversational Artificial Intelligence–Based Web Application for Medical Consultations: Prototype Study”

**DOI:** 10.2196/83217

**Published:** 2025-10-15

**Authors:** Anonymous

**Keywords:** artificial intelligence, ChatGPT, chatbots, conversational agent, machine learning

*This is the peer-review report for “Development of a Conversational Artificial Intelligence–Based Web Application for Medical Consultations: Prototype Study.”*

## Round 1 Review

### Specific Comments

#### Major Comments

1. I was really liking the idea of this paper [1] and read it with great interest, but perhaps I misunderstood—I was hoping it was a chatbot that would actually give me a diagnosis (eg, once I input an image of my retina or have some conversation with it) rather than just referring me to a specialist (which would be appropriate in some cases). Please correct my understanding if I am wrong.
2. From the initial paper idea, I got the feeling that it was going to be an app where I could start uploading images of X-rays and the chatbot, using its models, would start to tell me something about the image; instead, it seems from the example figures that all it is doing is telling the user that this is an X-ray image and to contact an expert. I am not sure how this is even useful? Perhaps I read the paper in haste and am lacking understanding. I would suggest showcasing a full conversation from each of your areas (X-rays, diabetes, etc), with a full screen capture of the conversation, showing an image uploaded and ending with a diagnosis (if indeed that is what your bot is capable of).
3. Figures 2-5 do not really give me any picture of what is going on, they just reaffirm what I thought; that is, that the bot is not actually giving any information except recognizing what type of image it is, then referring the user to a consultant? Is that correct? You really need to put some nice figures of your full flow and architecture, not too complex, but the ones you show do not really, in my opinion, provide the reader with any real value. As a reader, I want to see what it is you have done, and as a technical person who wants to replicate your work, I would want to see your architecture in diagram form, as well as a proper flowchart of some sort (again, no need to be complex, but to a high standard as you normally see in leading journals) outlining exactly what the flow is; this should correspond to the actual app screen captures so readers can see exactly what your app does.
4. Overall summary: Having worked on and researched chatbots, I read this with great interest but, as per my comments, I am a little confused, as it seems this bot simply refers the user to a person after recognizing an image as an X-ray, for example. I was under the impression from the content or was half expecting the ability to input an image, be that of an X-ray or retina, and it would start giving me some diagnostic information or the like.

#### Minor Comments

1. The manuscript is written in some places more like a personal blog; this should be changed and be more in line with how journal articles are written.
2. Many grammatical errors and some spelling mistypes, such as writing “chess” instead of “chest”; generally, it needs to be professionally proofread and the language tidied.

### Round 2 Review

#### General Comments

The author has given a response to each point and I am satisfied.
